# Genome-wide patterns of differentiation over space and time in the Queensland fruit fly

**DOI:** 10.1038/s41598-020-67397-5

**Published:** 2020-07-01

**Authors:** Ángel-David Popa-Báez, Renee Catullo, Siu Fai Lee, Heng Lin Yeap, Roslyn G. Mourant, Marianne Frommer, John A. Sved, Emily C. Cameron, Owain R. Edwards, Phillip W. Taylor, John G. Oakeshott

**Affiliations:** 1 Applied BioSciences, Macquarie University, Sydney, NSW 2109 Australia; 2 CSIRO Land and Water, Black Mountain, Canberra, ACT 2601 Australia; 3 Centre for Biodiversity Analysis, Ecology and Evolution, Australian National University, Canberra, ACT 2601 Australia; 4 School of Biological, Earth and Environmental Sciences, University of New South Wales, Sydney, NSW 2052 Australia; 5 Faculty of Health and Medicine, University of Newcastle, Callaghan, NSW 2308 Australia

**Keywords:** Evolutionary biology, Genetic hybridization, Genomics, Population genetics

## Abstract

The Queensland fruit fly, *Bactrocera tryoni*, is a major pest of Australian horticulture which has expanded its range in association with the spread of horticulture over the last ~ 150 years. Its distribution in northern Australia overlaps that of another fruit fly pest to which some authors accord full species status, *Bactrocera aquilonis*. We have used reduced representation genome-wide sequencing to genotype 359 individuals taken from 35 populations from across the current range of the two taxa, plus a further 73 individuals from six of those populations collected 15–22 years earlier. We find significant population differentiation along an east–west transect across northern Australia which likely reflects limited but bidirectional gene flow between the two taxa. The southward expansion of *B. tryoni* has led to relatively little genetic differentiation, and most of it is associated with a move into previously marginal inland habitats. Two disjunct populations elsewhere in Australia and three on Melanesian islands are each clearly differentiated from all others, with data strongly supporting establishment from relatively few founders and significant isolation subsequently. Resequencing of historical samples from one of the disjunct Australian populations shows that its genetic profile has changed little over a 15-year period, while the Melanesian data suggest a succession of *‘island hopping’* events with progressive reductions in genetic diversity. We discuss our results in relation to the control of *B. tryoni* and as a model for understanding the genetics of invasion and hybridisation processes.

## Introduction

The Queensland fruit fly (*Bactrocera tryoni* Froggatt, 1897; 'Qfly') is both a major pest of horticulture in Australia and some Pacific islands and a useful model for understanding genetic processes associated with range expansion and invasion^1^. Qfly was originally confined to the relatively wet tropical and sub-tropical east coast of Queensland (QLD) and the north eastern coast of New South Wales (NSW), with populations in the northern-most regions of the Northern Territory (NT) (nNT) and northern Western Australia (nWA) recognised by some authors as a distinct species, the Northern Territory fruit fly (NTfly, *B. aquilonis*)^2–4^. Over the last ~ 150 years, however, *B. tryoni* has expanded southward in association with the development of horticultural industries^5^. No infestations of Qfly were reported in the Sydney area (latitude ~ 33°5′ South) before the twentieth century^6^, but by the 1960s Qfly were found in Gippsland in Victoria (latitude ~ 37°5′ South)^7^. Later expansions included incursions further inland in QLD, NT and southern Australia^4^, with opportunistic invasions into New Caledonia, French Polynesia, Pitcairn Island and Cook Island in the Pacific^1^. Given increasing trade and horticultural activities in Australia and the region, climate modelling suggests a high likelihood of further expansion, under both current and future climate scenarios^8,9^.

Phenological work on Qfly in its native range suggests that host availability, as well as climate, are principal determinants of seasonal abundance, with rapid population increases in spring and summer and substantial decreases in autumn and winter^10^. This seasonal host availability effect is consistent with the range expansion which has occurred post-European settlement, and the associated introduction of new host crops. Many of the new crops depend on irrigation, and Yonow and Sutherst^8^ found the presence of irrigation to be a strong predictor of Qfly presence in northern, eastern and southern Australia. Several studies have reported strong plastic responses to low temperature in southerly populations^11,12^, as well as heavy dependence on overwintering refugia^13,14^, but there is little evidence for inherited geographic variation in cold resistance^15^. There is heritable ecotypic variation in heat and desiccation resistance, but these two traits are not strongly correlated with each other, or with latitude, suggesting complex patterns of geographic differentiation^15^.

Broadly consistent with all the distributional and ecological data, early population genetic studies with microsatellite markers indicated significant genetic differences across the species range^16,17^. In coastal eastern Australia there was an essentially panmictic population in the central eastern coast of Queensland (Rockhampton-Brisbane) with some differentiation to the north and south. Moreover there was also a progressive divergence westward along the north coast into the northern parts of the Northern Territory and northern Western Australia (nNT/nWA). An apparently distinct and potentially isolated population in central Australia was also identified^4,17^. As noted, several authors have classified the nNT/nWA populations as *B. aquilonis*, which is mainly distinguished from *B. tryoni* by a slightly paler thorax and abdomen, and more pointed aculeus on the ovipositor^2,3,18–20^. However, Drew and Lambert^19^ noted that the differences in colouration ‘are so slight as to be of questionable value for species-level discrimination’, and Wang et al.^21^ reported a Nei’s genetic distance of just 0.014 between the two taxa based on microsatellites. Cameron et al.^17^ argued that the nNT/nWA populations are simply progressively extreme samples of an essentially continuous clinal pattern of genetic differences from the east coast populations. There is as yet no genetic data on the island populations in the South Pacific but a specific succession of *‘island hopping’* invasions has been proposed based on the dates of the first records on those islands; Qfly was first reported in New Caledonia and the Loyalty Islands around 1969, in French Polynesia a year later, and subsequently in Cook Islands (albeit it was later eradicated from the latter)^22^.

The present study explores Qfly/NTfly population structure from across the known geographic range using over two thousand single nucleotide polymorphisms (SNPs) generated from genome-wide reduced representation resequencing. We examine both the spatial and temporal dimensions of the invasion process within Australia by sampling expansions southwards and inland and sampling and resampling from the centre and margins of the earlier range over a 15–22-year interval. Additionally, we characterise extant Pacific island populations to test the *‘island hopping’* hypothesis and investigate the genetics of disjunct as opposed to continuous spatial range expansions. Finally, we discuss the relevance of our results to the ecotypic variation, future distribution and management of the Qfly.

## Materials and methods

### Insect sampling

**Figure 1:**
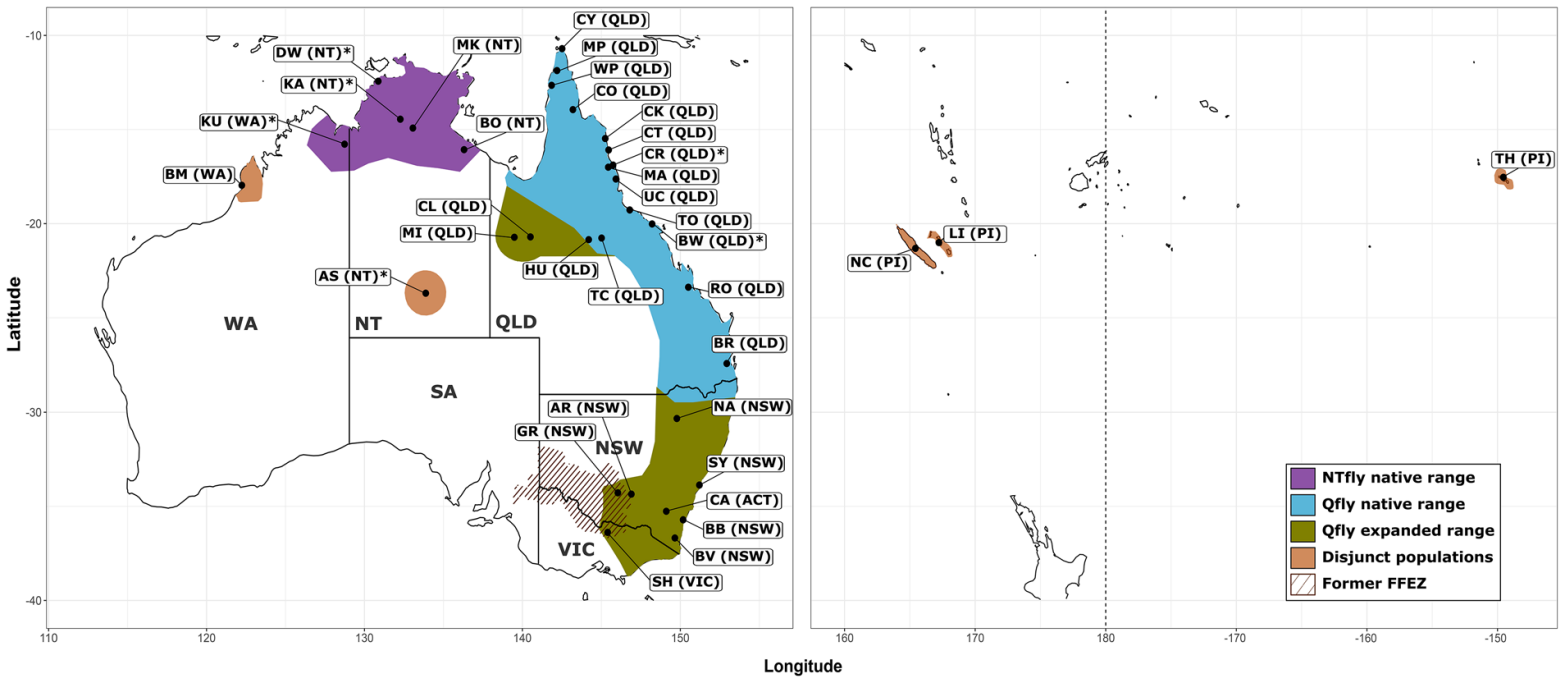
Geographic distribution of the Qfly and NTfly and our sampling sites. Distribution of the Australian populations within the Qfly native, expanded range and disjunct populations, our 35 collections sites and the former Fruit Fly Exclusion Zone (FFEZ). *AS* Alice Springs (Northern Territory, NT), *AR* Ardlethan (New South Wales, NSW), *BB* Batemans Bay (NSW), *BV* Bega Valley (NSW), *BO* Borroloola (NT), *BW* Bowen (Queensland, QLD), *BR* Brisbane (QLD), *BM* Broome (Western Australia, WA), *CR* Cairns (QLD), *CA* Canberra (Australian Capital Territory, ACT), *CT* Cape Tribulation (QLD), *CY* Cape York (QLD), *CL* Cloncurry (QLD), *CO* Coen (QLD), *CK* Cooktown (QLD), *DW* Darwin (NT), *GR* Griffith (NSW), *HU* Hughenden (QLD), *KA* Katherine (NT), *KU* Kununurra (WA), *LI* Loyalty Island (Pacific Islands, PI), *MP* Mapoon (QLD), *MA* Mareeba (QLD), *MK* Mataranka (NT), *MI* Mt Isa (QLD), *NA* Narrabri (NSW), *NC* New Caledonia (PI), *RO* Rockhampton (QLD), *SH* Shepparton (Victoria, VIC), *SY* Sydney (NSW), *TH* Tahiti (PI), *TC* Torrens Creek (QLD), *TO* Townsville (QLD), *UC* Utchee Creek (QLD), *WP* Weipa (QLD). Asterisk denote localities sampled in both 2015–2018 and 1994–2003.

We sampled a total of 368 individuals from 35 localities between 2015 and 2018, and for six locations we also had a total of 83 samples from 1994 to 2003 courtesy of the earlier microsatellite-based studies^4, 16^. Some flies were collected as adults from lure traps (BioTrap, Victoria, Australia) and then preserved in 100% ethanol. Others were collected as larvae from infested fruits, reared through to adults as part of the previous studies^15^ and then frozen at − 80 °C. Between four and 20 individuals were analysed per collection. Six *B. neohumeralis* (a closely related sister species in the tryoni complex) individuals which were used as an out-group in one analysis were collected from infested fruits from Cape Tribulation (16°08′ South), Brisbane (27°41′ South) and Mareeba (17°00′ South) and processed as per the Qfly and NTfly samples above. All flies were identified morphologically according to the species descriptions in Drew^20^, except that, like many of the previous authors, we could not consistently distinguish nNT/nWA flies from eastern states Qfly (although concurring that some of the former were somewhat paler). Figure 1 and Table 1 summarise all the populations analysed. (The maps in Fig. 1 and subsequent figures were prepare using *ggmap*^23^ and *ggplot2*^24^ in R version 3.6.1 (https://www.r-project.org/)^25^, with final edits in Inkscape 0.92.4, https://inkscape.org/).

**Table 1:** Qfly populations studied across all datasets. Year of establishment is according to earlier records in the published literature.

Population	Abbr.	State	Latitude	Longitude	Year of collection	Taxon	Female	Male	Established	Dataset	References
Borroloola	BO	NT	− 16.07	136.31	2017	NTfly	–	8	~ 1961	N	^4, 7^
Kununurra^a^	KU	WA	− 15.78	128.74	2015	NTfly	2	8	~ 1961	N	^4, 7^
Mataranka	MK	NT	− 14.92	133.07	2017	NTfly	–	8	~ 1961	N	^4, 7^
Katherine^a^	KA	NT	− 14.45	132.27	2017	NTfly	–	8	~ 1961	N	^4, 7^
Darwin^a^	DA	NT	− 12.43	130.87	2016	NTfly	1	6	~ 1961	N	^4, 7^
Brisbane^a^	BR	QLD	− 27.41	152.94	2016–2017	Qfly	7	13	< 1788	N	^4, 7^
Rockhampton	RO	QLD	− 23.38	150.51	2016	Qfly	6	6	< 1788	N	^4, 7^
Hughenden	HU	QLD	− 20.85	144.20	2017	Qfly	–	8	< 1788	N	^4, 7^
Torrens Creek	TC	QLD	− 20.77	145.02	2017	Qfly	–	8	< 1788	N	^4, 7^
Bowen	BW	QLD	− 20.02	148.22	2018	Qfly	–	8	< 1788	N	^4, 7^
Townsville	TO	QLD	− 19.28	146.80	2018	Qfly	–	8	< 1788	N	^4, 7^
Utchee Creek	UC	QLD	− 17.63	145.92	2017	Qfly	1	8	< 1788	N	^4, 7^
Mareeba	MA	QLD	− 17.00	145.44	2016–2017	Qfly	8	13	< 1788	N	^4, 7^
Cairns^a^	CR	QLD	− 16.89	145.74	2018	Qfly	–	4	< 1788	N	^4, 7^
Cape Tribulation	CT	QLD	− 16.09	145.46	2016–2018	Qfly	–	16	< 1788	N	^4, 7^
Cooktown	CK	QLD	− 15.48	145.25	2018	Qfly	–	8	< 1788	N	^4, 7^
Coen	CO	QLD	− 13.94	143.20	2018	Qfly	–	8	< 1788	N	^4, 7^
Weipa	WP	QLD	− 12.65	141.85	2018	Qfly	–	5	< 1788	N	^4, 7^
Mapoon	MP	QLD	− 11.87	142.19	2018	Qfly	–	8	< 1788	N	^4, 7^
Cape York	CY	QLD	− 10.70	142.51	2018	Qfly	–	11	< 1788	N	^4, 7^
Mt Isa	MI	QLD	− 20.73	139.49	2017	Qfly	–	8	> 1963	N + E	^4, 7^
Cloncurry	CL	QLD	− 20.71	140.51	2017	Qfly	–	8	> 1963	N + E	^4, 7^
Narrabri	NA	NSW	− 30.33	149.78	2017	Qfly	1	8	> 1907	N + E	^4, 7^
Sydney	SY	NSW	− 33.87	151.21	2016	Qfly	5	9	> 1,890	N + E	^4, 7^
Ardlethan	AR	NSW	− 34.35	146.90	2016	Qfly	–	8	> 1907	N + E	^4, 7^
Griffith	GR	NSW	− 34.28	146.05	2017	Qfly	1	8	> 1907	N + E	^4, 7^
Canberra	CA	ACT	− 35.40	149.10	2016	Qfly	6	11	> 1907	N + E	^4, 7^
Batemans Bay	BB	NSW	− 35.71	150.18	2017	Qfly	1	8	> 1907	N + E	^4, 7^
Bega Valley	BV	NSW	− 36.62	149.97	2016–2017	Qfly	4	9	> 1907	N + E	^4, 7^
Shepparton	SH	VIC	− 36.38	145.40	2016	Qfly	1	8	> 1907	N + E	^4, 7^
Broome	BM	WA	− 17.96	122.24	2015	NTfly	2	10	> 1963	N + E + D	^3, 7^
Alice Springs^a^	AS	NT	− 23.70	133.88	2016–2017	Qfly	10	19	~ 1980	N + E + D	^4^
New Caledonia	NC	PI	− 21.31	165.42	2018	Qfly	–	10	~ 1969	N + E + D	^22^
Loyalty Island	LI	PI	− 21.01	167.22	2018	Qfly	–	10	~ 1969	N + E + D	^22^
Tahiti	TH	PI	− 17.56	− 149.56	2018	Qfly	–	8	~ 1970	N + E + D	^22^
**1994 to 2003 collections**											
Kununurra	KU	WA	− 15.78	128.74	2000	NTfly	–	8	~ 1961	Temporal	^4, 7, 16, 17^
Katherine	KA	NT	− 14.45	132.27	2002	NTfly	–	5	~ 1961	Temporal	^4, 7, 16, 17^
Darwin	DA	NT	− 12.43	130.87	1994, 1999, 2002, 2003	NTfly	1	28	~ 1961	Temporal	^4, 7, 16, 17^
Brisbane	BR	QLD	− 27.41	152.94	2001	Qfly	–	8	< 1788	Temporal	^4, 5, 7, 16, 17^
Cairns	CR	QLD	− 16.89	145.74	2001	Qfly	–	11	< 1788	Temporal	^4, 7, 16, 17^
Alice Springs	AS	NT	− 23.70	133.88	1999–2001	Qfly	–	22	~ 1980	Temporal	^4, 7, 16, 17^

^a^Populations used in the temporal comparison analysis. *ACT* Australian Capital Territory, *NSW* New South Wales, *NT* Northern Territory, *PI* Pacific Island, *QLD* Queensland, *VIC* Victoria, *WA* Western Australia, *NTfly* Northern Territory fruit fly.

### DNA extraction, sequencing and SNP filtering

Individual flies were transferred from ethanol or − 80 °C storage into 96-well plates at − 20 °C and sent to Diversity Arrays Technology Pty Ltd (Canberra, Australia) for DNA extraction as per Kilian et al.^26^. *Pst*I and *Sph*I restriction enzyme digestion, ligation of < 200 bp fragments to a bar coding adapter, PCR amplification, and Illumina HiSeq2500 sequencing were completed following the DArTseq protocol of Georges et al.^27^. SNPs were called from the resulting sequence reads using the DArT analytical pipeline as described by Georges et al.^27^ and the resulting dataset was read into R, together with associated metadata, using the helper functions of the *dartR* R package^28^. Filtering of SNPs involved excluding those which were (a) not scored consistently in two replicate runs of the protocol (see George et al.^27^ for details), (b) had < 10 × or > 250 × read depth or c) a call rate < 90% (i.e. SNPs not found in ≥ 10% of the individuals). Filtering of individuals then involved excluding those with a call rate < 90% (i.e. individuals with ≥ 10% of the SNPs missing). Finally, we only retained one randomly chosen SNP from any sequenced tag containing multiple SNPs. After filtering, the data from nine individuals in the N + E + D dataset (see below) were excluded, as were 23 individuals from the temporal dataset (the nine above plus four more from the 2015–2018 samples and ten from the 1994–2013 samples).

### Data structure

The analyses below were structured around four sets of samples:

1. Native range (N) populations: the 20 2015–2018 collections in the Native Qfly/NTfly range.
2. Expansion (E) populations: samples from 10 populations in the essentially contiguous expansion southward and inland.
3. Disjunct (D) populations: samples from Broome, Alice Springs, and the Melanesian islands, and
4. Temporal data: samples from six locations, collected 15–22 years earlier (1994–2003) for the previous microsatellite studies, plus the 2015–2018 collections from the same locations.

Depending on the question, the analyses below were conducted on the three spatial datasets (N, N + E, N + E + D) or the temporal dataset.

### Population structure

The first of two tests for population structure we carried out on all datasets was a Discriminant Analysis of Principal Components (DAPC)^29^, which was implemented in the R package “adegenet”^30^ and used the localities sampled as priors for populations. The optimal number of Principal Components (PCs) to be retained was selected using alpha score optimisation with the *optim.a.score* function in the “adegenet” package, retaining 83, 77, 51, and 12 PCs from the N, N + E, N + E + D and temporal datasets, respectively (Fig. S1). The Bayesian information criterion (BIC) was used to identify the best number of discriminant functions to represent the data. We retained two discriminant functions for the N and N + E, and five and three discriminant functions for the N + E + D and temporal datasets, respectively (see Fig. S1).

The second test for population structure used sparse, non-negative matrix factorisation (sNMF^31^) to estimate individual fly admixture coefficients. This involves an algorithm which is similar to the Bayesian clustering program structure^32^ but uses non-negative matrix factorisation and least-square optimisation to facilitate working with large datasets. The R package LEA^31^ implements this algorithm in the *snmf* function. The SNP data were converted first into a Structure-like input format with the *gl2faststructure* function from the dartR package and then into the required format for the *snmf* function with the helper function *struct2geno* from the LEA package. The *snmf* algorithm was run with K values for all the datasets, with 100 repetitions per K value and other options set to default values in all cases. The value of K that best explained the results was selected using the cross-entropy criterion as detailed by the LEA package authors^31^ (Fig. S2).

### Isolation by distance

We conducted two analyses to test for any isolation by distance in the N + E dataset. First we ran a Mantel test of the linearised genetic distances between populations (Nei’s D/1 − Nei’s D, calculated using the R package StAMPP^33^) against the geographical distance (Euclidean distance, calculated using the distance function in the R package vegan^34^). Secondly, we conducted a redundancy analysis (RDA) of allele frequencies against polynomial functions of latitude and longitude (maximum power two, calculated with *poly* and forward selected with *ordistep* from the vegan package^34^) in this dataset as per Meirmans^35^. The overall model and specific functions within it were tested for significance using the *anova.cca* function from the vegan package^34^ with 1,000 permutations for each test. We then multiplied the percentage of variance accounted for by the latitude and longitude of each population (i.e. the constrained variance of the RDA) by the overall Nei’s D for all populations to obtain the percentage of the total genetic variation that is explained by the spatial variables^35,36^.

### Phylogenetics

We investigated the origins of the contiguous expansion (E) and disjunct invasion (D) populations by inferring a population tree from the N + E + D dataset using TreeMix^37^. TreeMix was preferred over traditional phylogenetic approaches because of our primary interest in tracing the origin of the range expansion and disjunct (E and D) populations. We created an input file of allele frequencies for every contemporary sample according to the TreeMix manual. We then built a maximum likelihood tree from the allele frequencies using the default 500 SNP block setting, rooting the trees with *B. neohumeralis* and visualising them with the R script provided with the software^38^.

### Fixed difference and other genetic parameters

We tested for fixed differences between populations in the N + E + D dataset using the *gl.collapse.recursive* function in the dartR package^28^. This method sums the fixed differences between each pair of populations and combines the members of each pair which do not have a significant number of fixed differences, where the number of differences expected due to sampling error is estimated from a thousand bootstrapping runs with the *gl.collapse.pval* function. The process is repeated until no further non-significant pairs are found^28^. The resulting set of collapsed populations can be thought of as diagnostic operational taxonomic units (OTUs) between which there is strong support for an absence of recent gene flow.

We also estimated genetic diversity summary statistics (allele richness, Shannon diversity, and heterozygosity) for all populations, using the formulae of Sherwin et al.^39^ implemented in the *gl.diversity* function in the *dartR* package^28^. Additionally we calculated Fst between all populations in the N + E + D dataset with the *gl.fst.pop* function from the dartR package and tested their significance using 1,000 bootstrap replications.

### Temporal variation

The temporal dataset contained six pairs of samples from previous Qfly population genetic studies^4,16,17^ and contemporary populations collected from the same localities (Table 1). We first tested for overall differences due to the six localities and two sampling times in the genetic distances between the 12 samples (calculated with the helper function *dist* in R) using the *poppr.amova* function in the poppr package and simulations with 1,000 permutations generated with the *randtest* function^40^. We then screened for changes within each population pair over the 15–22 years by calculating Fst between the two temporal samples from each locality with the *gl.fst.pop* function from the dartR package and testing their significance using 1,000 bootstrap replications.

## Results

After quality filtering, the data comprised 2,785 SNPs for 359 individuals for the 2015–2018 samples for the spatial analyses and 2,344 SNPs (~ 58% of them also in the 2,785 above) from 138 individuals for the temporal analyses (i.e. the six populations scored on both the 2015–2018 and 1994–2003 datasets).

### Population structure in the native and expanded range

**Figure 2:**
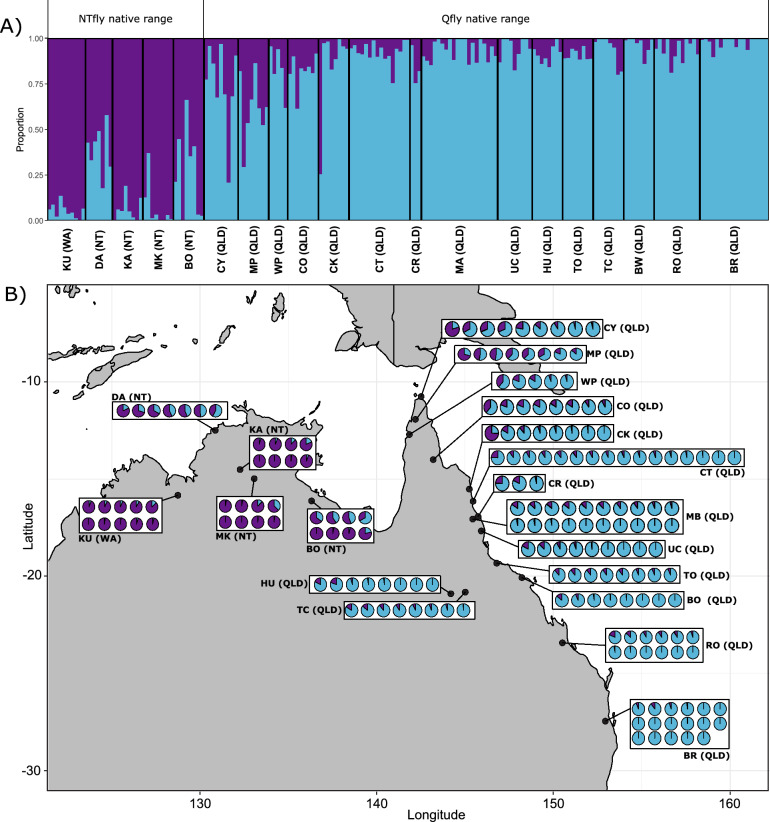
Analysis of Qfly and NTfly native range populations. The ancestry coefficients for 20 populations from the Qfly and NTfly native range are presented. (**A**) Estimates of admixture proportions inferred with sNMF for the Qfly and NTfly native dataset (*N*) with the best supported number of ancestral populations (K = 2). (**B**) Geographical distribution of the Qfly and NTfly within its native range. Pie charts represent the admixture proportion of every individual for each population estimated with sNMF. Population abbreviations are as per Fig. 1.

We used a combination of clustering (DAPC) and admixture coefficients (sNMF) analyses to test for differentiation among the 20 populations we sampled from the Qfly/NTfly native range (i.e., dataset N). DA1 in the DAPC analysis accounted for 31.5% of the variance and separated most of the nNT/nWA populations from the QLD populations, with Darwin (DA) and Borroloola (BO) as intermediates. DA2, accounting for 16% of the variance, separated Darwin populations from the others (Fig. S3). The cross-entropy algorithm in sNMF yielded the lowest cross-entropy for a K of two, with less support for other values of K (3–10; Fig. S2). Similar to the DAPC results, sNMF differentiated the NTfly and Qfly populations, but it also showed considerable individual variation within them (Fig. 2). Some Darwin and Borroloola individuals showed 50% or more QLD ancestry, whereas Kununurra (KU) individuals shared little ancestry with the east coast populations. Notably Kununurra is furthest from the east coast among these populations while Borroloola is closest, and Darwin is a major sea- and airport city, with significant trade and travel links to the east coast. Among the QLD samples, the more northerly coastal populations (Cape York, Mapoon, Coen, Weipa, Cook Town and Cairns, i.e. CY, MP, CO, WP, CT, CR) included individuals showing significant nNT/nWA ancestry, whereas individuals in the most southerly population, Brisbane (BR), had the lowest level of admixture. The two inland QLD populations, Hughenden (HU) and Torrens Creek (TC), are relatively isolated geographically and, while relatively northerly, they also showed relatively little nNT/nWA ancestry.

**Figure 3:**
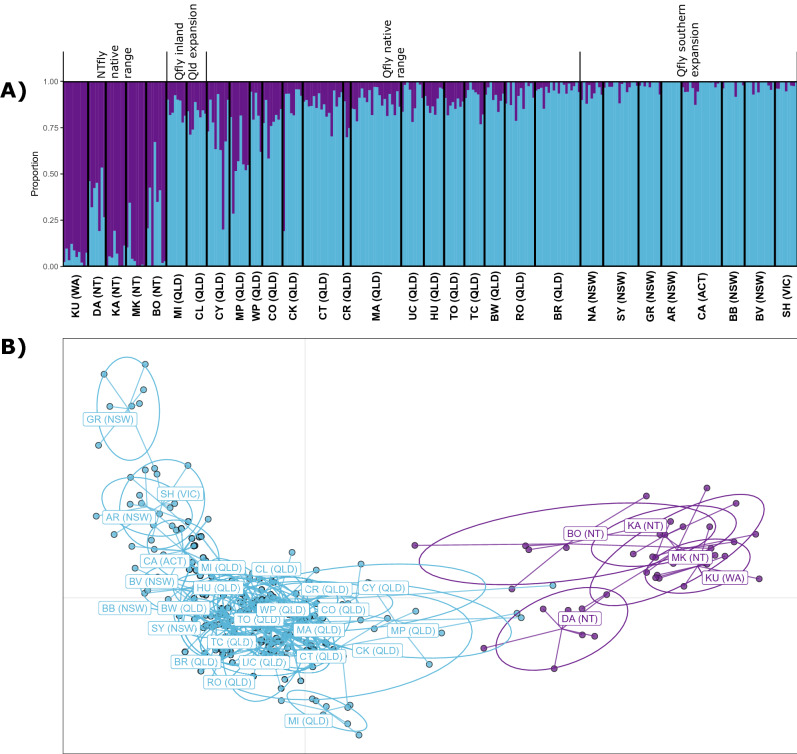
Analysis of the Qfly expanded range populations. (**A**) Estimates of admixture proportions inferred with sNMF for the Qfly and NTfly native and Qfly expanded range dataset (N + E) with the best supported number of ancestral populations (K = 2). (**B**) DAPC of individuals within the Qfly and NTfly native and Qfly expanded dataset. Colours are representative of the admixture proportions of every individual estimated with sNMF. Population abbreviations are as per Fig. 1.

The sNMF analysis including the southerly and north-western QLD populations (i.e., the N + E dataset) found a K value of two yielded the lowest cross-entropy (Fig. S2) and showed the southern populations had mainly QLD and little or no NTfly ancestry (Fig. 3a). However the DAPC analysis also showed that the three most inland southerly populations, Griffith, Ardlethan and Shepparton (GR, AR and SH respectively) were somewhat further separated from the nNT/nWA group than the rest (Fig. 3b). The DAPC analysis also showed the two most inland northern populations, Mt. Isa (MI) and Cloncurry (CL), had mostly QLD ancestry but with greater admixture with nNT/nWA than many of the populations to their east (Fig. 3).

Consistent with a genetic structure suggestive of isolation by distance, a Mantel test of genetic vs geographic distance between all pairs of populations in the N + E dataset yielded a significant positive correlation with geographic distance (Mantel’s r = 0.37, P = 0.001). However, the redundancy analysis (RDA; Fig. S4) showed this only explained 7.6% of the population differences, equivalent to a value of Nei’s D of 0.01 (F_7, 283_ = 3.31, P = 0.001). Most of the spatial effect was related to longitude (RDA1 = 61%, F_1, 283_ = 14.17, P < 0.001), which is consistent with the distinction between the QLD and nNT/nWA ancestry groups. However, the low overall explanatory value of distance indicates there is also significant gene flow unrelated to distance and probably facilitated by horticultural trade among the native range and contiguous expansion populations.

### Origins of the disjunct populations

**Figure 4:**
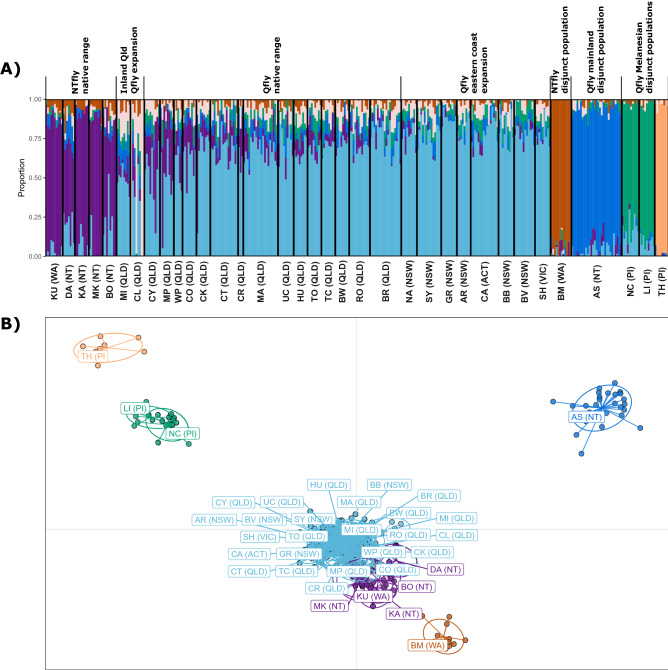
Analysis of the disjunct populations. (**A**) Estimates of admixture proportions inferred with sNMF for the native, expanded, and disjunct dataset (N + E + D) with the best supported number of ancestral populations (K = 6). (**B**) DAPC of individuals within the native, expanded, and disjunct dataset (N + E + D). Colours are representative of the admixture proportions of every individual estimated with sNMF. Population abbreviations are as per Fig. 1. Also see Fig. S5 for an IQ-tree phylogeny of all individuals in the expansion and disjunct datasets.

DAPC and sNMF analyses including all 35 contemporary (i.e. N + E + D) samples identified the five geographically disjunct populations as genetically distinct (Fig. 4). The three Pacific Island populations were separated on both DA1 and DA2, with New Caledonia (NC) and Loyalty Island (LI) most similar, and with Tahiti (TH) as their next nearest neighbour, although Tahiti was even more divergent from all mainland populations. The two disjunct Australian populations, Broome (BM) and Alice Springs (AS), were also well separated from the other Australian samples in the DAPC plot, but they were also well separated from each other. The sNMF analysis identified the lowest cross-entropy for a K value of five (Fig. S2), corresponding to ancestry groupings of QLD and nNT/nWA as identified above, as well as the Pacific Islands (as a cluster), Alice Springs and Broome.

**Figure 5:**
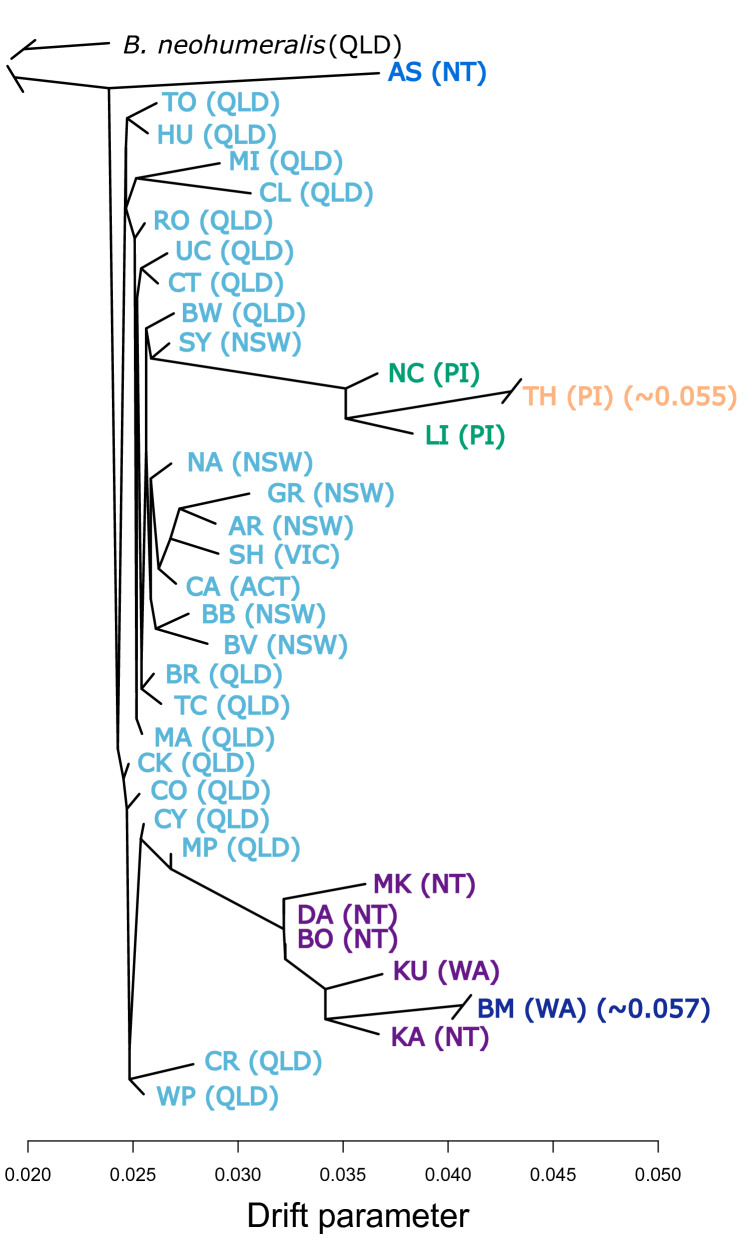
TreeMix analysis for the origin of the contiguous expansion and disjunct populations. The maximum-likelihood tree inferred with TreeMix depicts phylogenetic relationships of the 35 populations in the N + E + D dataset with *B. neohumeralis* as an outgroup. Colours are representative of the admixture proportions of every individual estimated with sNMF, as in Fig. 4. Population abbreviations are as per Fig. 1.

A TreeMix maximum likelihood tree of all 35 contemporary samples, using *B. neohumeralis* as the outgroup (Fig. 5), had little resolution (i.e. low branch length/drift parameters) among the native range and expansion populations (nor did an IQ-tree maximum likelihood tree of all N + E + D individuals; Fig. S5). TreeMix placed the northern nNT/nWA populations together with one another but separated from the QLD and expansion populations by somewhat higher drift parameters. The five disjunct populations were all separated by relatively high drift parameters from all the other populations above. Broome apparently derived from its geographically most proximal population, the nNT/nWA group, although the source of the Alice Springs population remains unclear (Fig. 5; Fig. S5). Finally, the three Pacific Island populations were closest relatives of each other and joined by a long branch to populations from the Australian east coast, with Tahiti having drifted further than the other two.

The fixed difference analysis, testing for populations with high differentiation in the contemporary samples (N + E + D), found no populations or groups of populations were distinguished by a statistically significant number of fixed differences (OUT = 1, P > 0.05, Table S1). Shannon diversity statistics and heterozygosity values were also relatively low, the latter particularly for the disjunct populations (Table S2). Fst values were likewise low across all pairwise comparisons (mean Fst value of 0.09), albeit slightly higher for comparisons involving disjunct populations (Table S3). These results confirm the relatively high levels of genetic similarity across the native range and expansion populations, and further suggest that the disjunct populations are recent invasions from small founding populations.

### Temporal variation

**Figure 6:**
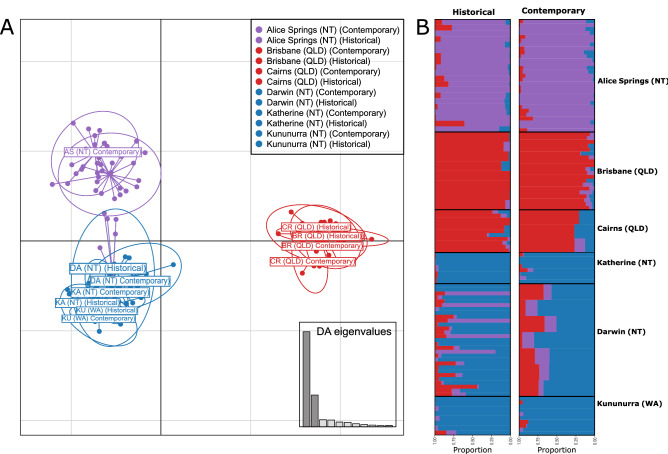
Analysis of temporal variation in six Qfly populations. (**A**) Estimates of admixture proportions inferred with sNMF for the historical and contemporary populations groups with the best supported number of ancestral populations (K = 3). (**B**) DAPC of individuals from the historical and contemporary population groups. Colours are representative of the admixture proportions of every individual estimated with sNMF. Population abbreviations are as per Fig. 1.

The six populations from which we had both contemporary and 1994–2003 collections included three nNT/nWA and two QLD ancestry groups, plus the disjunct Alice Springs population. DAPC and sNMF analyses of the 138 individuals whose sequences survived the filtering in these six population pairs (Fig. 6) showed little change in population structure and differentiation over the last 15–22 years. An AMOVA indicated that 91% of the variance was explained by geographic differentiation ($\phi =$ 0.08, P < 0.0001) but only about 1% by temporal changes, although the latter was still statistically significant ($\phi =$ 0.01, P < 0.001). Such temporal changes as were found were largely in Darwin (Fst = 0.017, P < 0.001) and Cairns (Fst = 0.014, P < 0.001); in the latter the ancestry group principally associated with Alice Springs was more common in the older samples. More notably, both the broad nNT/nWA vs QLD and Alice Springs vs the rest distinctions were seen in both sampling periods. While the fixed difference analyses and Shannon diversity statistics suggest relatively high levels of genetic similarity among contiguous populations and relatively recent origins of the disjunct populations, the temporal data thus indicate that the nNT/nWA vs QLD distinction and the uniqueness of the Alice Springs population have changed little in the last 15–22 years.

## Discussion

There is little genetic differentiation across the native and expanded Qfly range down the east coast of Australia, but larger differences are evident along an east/west transect across the north of Australia. These results concur with the pattern of differences obtained in earlier allozyme^19^ and microsatellite studies^17^. As noted, some authors have suggested the existence of two distinct taxa in northern Australia, *B. tryoni* in the east and *B. aquilonis* in the northern parts of the Northern Territory and northwest Western Australia. Cameron et al.^17^ suggested their microsatellite data could reflect recent gene flow between those taxa, aided by increased production and trade of horticultural commodities across northern Australia^18^. The fact that our Darwin samples show more similarity to the east coast populations than do other northern NT populations supports the notion that trade contributed to the introgression. The absence of significant numbers of fixed differences even between the most widely separated populations along the transect suggests that the taxonomic distinction might not have amounted to species status, at least in recent times. However, research on ecotypic variation^15^ did not include any minimally introgressed *B. aquilonis* (the most *B. aquilonis*-like collection in that study being Darwin), so it remains possible that the populations further west than those in our analysis are ecologically distinct from *B. tryoni.* There are several examples in the literature whereby insect species have retained ecotypic distinction in the face of quite high levels of genetic introgression^41,42^. The east/west transect covered here crosses a number of biogeographical barriers which have led to differentiation between closely related species of amphibians^43^, birds^44–46^, other insects^47,48^, mammals^49^ and plants^50^ across different geological periods^51,52^.

Populations from the southerly Qfly expansion down the east coast show little genetic differentiation from the north coast populations, except for progressively less evidence of nNT/nWA genotypes. This fits with an expectation^53, 54^ that these southerly coastal populations would have derived from the adjacent native Qfly range rather than the more distant nNT/nWA.

Although the differences are not large, the three more interior southern expansion populations, Griffith, Ardlethan and Shepparton, are all somewhat displaced from their coastal counterparts and more distinct from the nNT/nWA genotypes in the DAPC plot. These populations are in regions previously classified as ecologically marginal for the Qfly^8^, and are the closest in our sample to a region previously recognised as a Fruit Fly Exclusion Zone (FFEZ)^55^ (Fig. 1). The FFEZ covered the Murrumbidgee Irrigation Area of NSW and adjacent Riverland areas of South Australia, Victoria and NSW, but all except the South Australian Riverland lost its FFEZ designation in July 2013 because of the growing Qfly populations immediately adjacent to it, and increasingly frequent Qfly outbreaks within it^55^. Microsatellite studies showed that those incursions were generally from the adjacent populations^53,54,56^. Given our evidence that these adjacent populations show some differentiation from Qflies elsewhere, the intensive Sterile Insect Technique (SIT) control programmes now under way in what was the FFEZ^57,58^ may benefit from using the adjacent populations as source material for their mass release strains.

Our data for the three disjunct Melanesian populations support an *island-hopping* model in which flies migrated from the east coast of Australia into New Caledonia and Loyalty Island (~ 1,600 and 1,800 km east, respectively), which in turn became the source of migrants to the more distant Tahiti (~ 6,400 km further east)^22^. Thus our data show a progressive reduction of genetic diversity (Tables S2, S3) and increasing differentiation from their Australian ancestors (Fig. 4) during this process.

A similar loss of genetic diversity and clear differentiation is seen in one of the disjunct Australian populations, Broome, in the far northwest of Australia, but in this case the most likely source was the nNT/nWA population (~ 1,130 km east and separated by the Kimberley range). Our data did not identify the source of the other disjunct Australian population, Alice Springs, in central Australia. Notwithstanding its isolation it is not deficient in genetic diversity. This is evident in our temporal analysis, which shows the genetic composition of the Alice Springs population has changed very little in the 15 years between samples, suggesting its founding population was large enough to avoid bottleneck issues and there has been little immigration since.

Given that all five of the disjunct populations show a level of genetic differentiation (i.e., high drift parameter values) indicative of low gene flow with other populations, they could all be good candidates for local eradication programs based on SIT.

We can compare our evidence for genetic differentiation across the Qfly/NTfly range with the data of Popa-Báez et al.^15^ for ecotypic differentiation in relation to abiotic stress resistance. They only studied 12 populations, mostly from the east coast of Australia but also including Darwin, Alice Springs and Griffith. Darwin and Alice Springs were found to be distinguished by relatively high heat resistance, and Griffith by relatively high desiccation resistance, all of which is consistent with the genetic differentiation from the east coast populations evident in the present study. Among their ten east coast populations, however, Cape Tribulation (CT, their most northerly population) and Sydney (SY) were found to have relatively high desiccation resistance, and Sydney relatively high heat resistance as well, which would not have been predicted from our data. Furthermore, a separate sample of the Sydney population was collected and tested two years later for desiccation resistance and again found to have relatively high resistance, suggesting it was a stable, inherited feature of the population^15^. While our spatial analysis is based on over 2,700 SNPs taken randomly from across the genome, it can clearly miss phenotypically important genetic differences whose distribution across the species range departs from those seen from the overall trends evident in the summary statistics from our data.

It is interesting to compare the results presented in this study and Popa-Báez et al.^15^ with those of Qin et al.^59^ on *B. dorsalis*, another widely distributed tephritid pest. They screened for differences in four taxa previously recognised as different species from a wide range of locations in Africa, Asia and Hawaii, using microsatellite and morphometric (wing length) analysis. Their microsatellite work revealed differences between the African and Hawaiian samples on one hand and the east Asian populations on the other, with only minor differences in wing length among their samples. In contrast, the stronger evidence of genetic differentiation and ecotypic variation evident in the current study and Popa-Báez et al.^15^ might in part reflect a greater power of our DArT data and the multiple stress response traits measured by Popa-Báez et al.^15^, but it could also indicate a stronger tendency for local differentiation in Qfly.

## Supplementary information

Supplementary Information.

## Data Availability

The SNPs data and scripts used in this study are available in the Figshare Population genomics of the Queensland fruit fly repository (https://doi.org/10.6084/m9.figshare.11390043.v1) and GitHub repository (https://github.com/Angel-Popa/Qfly_pop-gen), respectively.
