# Supplementary material for: Genome-wide patterns of differentiation over space and time in the Queensland fruit fly
Source: Sci Rep. 2020 Jul 1;10:10788. doi: 10.1038/s41598-020-67397-5 (PMC7329829; doi:10.1038/s41598-020-67397-5)
Supplement: Supplementary file 1 — Supplementary Information. [file 41598_2020_67397_MOESM1_ESM.pdf]

Supplementary figures and tables

Genome-wide patterns of differentiation over space and time in the Queensland fruit fly

Ángel-David. Popa-Báez †* [1,2], Renee Catullo † [2,3], Siu Fai Lee [1,2], Heng Lin Yeap [2], Roslyn G. Mourant [2], Marianne Frommer [4], John A. Sved [4], Emily C. Cameron [5], Owain R. Edwards [1,2], Phillip W. Taylor [1] and John G. Oakeshott [1,2]

[1] Applied BioSciences, Macquarie University, Sydney, NSW 2109, Australia.

[2] CSIRO Land and Water, Canberra, ACT 2601, Australia.

[3] Centre for Biodiversity Analysis, Ecology & Evolution, Australian National University, Canberra, ACT 2601, Australia.

[4] School of Biological, Earth and Environmental Sciences, University of New South Wales, Sydney, NSW 2052, Australia

[5] Faculty of Health and Medicine, University of Newcastle, Callaghan, NSW, 2308, Australia

*Corresponding Email: Angel.Popa@csiro.au

† These authors contributed equally to this manuscript.

Running title: Population genomics of the Queensland fruit fly

Table S1: Results of fixed differences analysis of populations in the N+E+D dataset. The lower triangle shows the number of fixed SNPs found between population pairs, and the upper triangle shows the p-value of each comparison. Significant comparisons are shown as red cells. The OUT column shows the results of the recursive fixed difference analysis indicating the lack of operational taxonomical units (OUTs) detected across all population pairs in the complete dataset.

	AS	AR	BB	BV	BO	BW	BR	BM	CR	CA	CT	CY	CL	CO	CK	DA	GR	HU	KA	KU	LI	MP	MA	MK	MI	NA	NC	RO	SH	SY	TH	TC	TO	UC	WP	OUT
**AS**		0.79	0.70	0.67	0.89	0.71	0.55	0.00	1.00	0.56	0.56	0.75	0.86	0.73	0.77	0.89	0.82	0.75	0.97	0.00	0.95	0.77	0.57	0.00	0.77	0.72	0.84	0.60	0.80	0.61	1.00	0.76	0.75	0.85	0.93	
**AR**	0		0.81	0.78	0.98	0.83	0.67	0.01	1.00	0.68	0.66	0.83	0.95	0.87	0.86	1.00	0.81	0.83	0.99	0.00	0.99	0.89	0.57	0.03	0.92	0.78	0.96	0.73	0.82	0.69	1.00	0.82	0.90	0.78	0.99	
**BB**	0	0		0.74	0.97	0.81	0.54	0.00	1.00	0.67	0.67	0.79	0.91	0.78	0.80	0.97	0.81	0.78	1.00	0.09	0.96	0.83	0.64	0.00	0.84	0.71	0.87	0.64	0.82	0.67	1.00	0.76	0.79	0.73	0.97	
**BV**	0	0	0		0.96	0.74	0.60	0.05	1.00	0.62	0.58	0.71	0.89	0.70	0.73	0.94	0.75	0.75	1.00	0.00	0.92	0.76	0.57	0.00	0.85	0.66	0.84	0.65	0.72	0.64	1.00	0.72	0.73	0.68	0.95	
**BO**	0	0	0	0		0.95	0.87	1.00	1.00	0.88	0.87	0.89	0.98	0.91	0.92	0.96	0.98	0.93	0.90	0.82	0.99	0.91	0.81	0.88	0.97	0.96	0.76	0.87	0.97	0.93	1.00	0.97	0.95	0.95	1.00	
**BW**	0	0	0	0	0		0.61	0.57	1.00	0.66	0.67	0.80	0.92	0.81	0.82	0.99	0.88	0.80	0.99	0.00	0.98	0.83	0.60	0.00	0.88	0.72	0.90	0.65	0.82	0.62	1.00	0.82	0.85	0.76	0.98	
**BR**	0	0	0	0	0	0		0.00	1.00	0.58	0.55	0.54	0.70	0.68	0.62	0.85	0.69	0.63	0.99	0.00	0.89	0.65	0.53	0.00	0.68	0.60	0.69	0.58	0.56	0.57	1.00	0.68	0.60	0.60	0.82	
**BM**	4	8	8	4	0	5	6		1.00	0.00	1.00	0.95	0.87	0.91	1.00	1.00	0.00	0.00	1.00	0.99	0.07	1.00	0.99	1.00	0.40	0.09	0.15	0.00	0.00	0.00	0.14	0.99	0.17	0.95	1.00	
**CR**	0	0	0	0	0	0	0	3		1.00	1.00	1.00	1.00	1.00	1.00	1.00	1.00	1.00	1.00	0.71	1.00	1.00	0.99	0.92	1.00	1.00	1.00	1.00	1.00	1.00	1.00	1.00	1.00	1.00	1.00	
**CA**	0	0	0	0	0	0	0	7	0		0.54	0.64	0.76	0.61	0.68	0.84	0.64	0.62	0.92	0.00	0.89	0.72	0.52	0.00	0.75	0.58	0.73	0.58	0.63	0.52	1.00	0.63	0.62	0.62	0.86	
**CT**	0	0	0	0	0	0	0	0	0	0		0.66	0.72	0.64	0.61	0.88	0.72	0.69	0.98	0.00	0.91	0.64	0.53	0.00	0.67	0.61	0.75	0.60	0.62	0.54	1.00	0.66	0.71	0.66	0.86	
**CY**	0	0	0	0	0	0	0	2	0	0	0		0.87	0.75	0.80	0.98	0.83	0.76	0.98	0.99	0.97	0.77	0.59	1.00	0.88	0.71	0.90	0.66	0.73	0.69	1.00	0.79	0.81	0.74	0.97	
**CL**	0	0	0	0	0	0	0	4	0	0	0	0		0.90	0.90	1.00	0.95	0.90	1.00	0.00	1.00	0.94	0.71	0.00	0.95	0.86	0.99	0.82	0.92	0.80	1.00	0.90	0.92	0.97	1.00	
**CO**	0	0	0	0	0	0	0	4	0	0	0	0	0		0.80	0.98	0.82	0.82	0.99	0.99	0.98	0.84	0.62	1.00	0.90	0.80	0.91	0.70	0.83	0.72	1.00	0.80	0.78	0.79	0.97	
**CK**	0	0	0	0	0	0	0	1	0	0	0	0	0	0		0.94	0.86	0.81	0.99	0.99	0.96	0.82	0.58	0.99	0.87	0.77	0.92	0.72	0.82	0.69	1.00	0.84	0.80	0.74	0.97	
**DA**	0	0	0	0	0	0	0	0	0	0	0	0	0	0	0		0.98	0.99	0.96	0.92	1.00	0.93	0.79	0.95	0.97	0.96	1.00	0.87	0.99	0.91	1.00	0.98	0.99	0.95	1.00	
**GR**	0	0	0	0	0	0	0	11	0	0	0	0	0	0	0	0		0.86	1.00	0.02	0.98	0.89	0.67	0.00	0.93	0.80	0.93	0.76	0.85	0.68	1.00	0.85	0.88	0.82	0.99	
**HU**	0	0	0	0	0	0	0	10	0	0	0	0	0	0	0	0	0		0.96	1.00	0.98	0.86	0.59	1.00	0.90	0.77	0.94	0.69	0.79	0.61	1.00	0.86	0.77	0.80	0.98	**1**
**KA**	0	2	1	0	0	1	0	0	3	1	0	0	0	0	0	0	1	1		0.78	0.87	0.97	0.96	0.83	0.95	0.99	1.00	0.98	0.86	0.91	1.00	0.80	1.00	0.99	1.00	
**KU**	6	6	4	4	0	7	4	0	7	4	6	0	6	0	0	0	5	0	0		0.00	0.99	0.00	0.82	1.00	0.06	0.14	1.00	0.00	0.00	0.76	0.99	0.02	1.00	0.00	
**LI**	0	0	0	0	1	0	0	9	0	0	0	0	0	0	0	0	0	0	4	7		0.98	0.88	0.00	0.99	0.97	0.76	0.93	0.98	0.89	1.00	0.97	0.98	0.97	1.00	
**MP**	0	0	0	0	0	0	0	0	0	0	0	0	0	0	0	0	0	0	0	0	0		0.62	0.99	0.90	0.80	0.92	0.76	0.87	0.70	1.00	0.86	0.87	0.84	0.98	
**MA**	0	0	0	0	0	0	0	0	0	0	0	0	0	0	0	0	0	0	0	4	0	0		0.00	0.66	0.56	0.67	0.52	0.58	0.56	1.00	0.62	0.59	0.61	0.74	
**MK**	7	6	6	5	0	7	5	0	7	5	7	0	7	0	0	0	7	0	0	0	9	0	5		1.00	0.00	0.18	1.00	0.00	0.00	0.76	1.00	0.00	1.00	0.00	
**MI**	0	0	0	0	0	0	0	5	0	0	0	0	0	0	0	0	0	0	1	0	0	0	0	0		0.87	0.95	0.74	0.86	0.68	1.00	0.88	0.90	0.86	0.99	
**NA**	0	0	0	0	0	0	0	7	0	0	0	0	0	0	0	0	0	0	1	4	0	0	0	6	0		0.85	0.65	0.77	0.65	1.00	0.76	0.80	0.73	0.97	
**NC**	0	0	0	0	1	0	0	10	0	0	0	0	0	0	0	0	0	0	2	5	0	0	0	6	0	0		0.73	0.94	0.73	1.00	0.93	0.92	0.89	1.00	
**RO**	0	0	0	0	0	0	0	5	0	0	0	0	0	0	0	0	0	0	0	0	0	0	0	0	0	0	0		0.78	0.61	1.00	0.71	0.65	0.65	0.90	
**SH**	0	0	0	0	0	0	0	9	0	0	0	0	0	0	0	0	0	0	2	6	0	0	0	6	0	0	0	0		0.68	1.00	0.80	0.85	0.81	0.98	
**SY**	0	0	0	0	0	0	0	6	0	0	0	0	0	0	0	0	0	0	1	4	0	0	0	5	0	0	0	0	0		1.00	0.66	0.69	0.64	0.92	
**TH**	0	0	0	0	1	0	0	17	1	0	0	1	1	0	0	0	1	0	5	8	0	1	0	9	3	1	0	0	0	0		1.00	1.00	1.00	1.00	
**TC**	0	0	0	0	0	0	0	3	0	0	0	0	0	0	0	0	0	0	2	1	0	0	0	0	0	0	0	0	0	0	0		0.82	0.79	0.98	
**TO**	0	0	0	0	0	0	0	7	0	0	0	0	0	0	0	0	0	0	0	4	0	0	0	5	0	0	0	0	0	0	0	0		0.82	0.98	
**UC**	0	0	0	0	0	0	0	4	0	0	0	0	0	0	0	0	0	0	1	0	0	0	0	0	0	0	0	0	0	0	0	0	0		0.98	
**WP**	0	0	0	0	0	0	0	1	0	0	0	0	0	0	0	0	0	0	0	6	0	0	0	7	0	0	0	0	0	0	2	0	0	0		

Table S2: Genetic diversity statistics for populations in the N+E+D dataset.

Populations	Abbr.	Allelic richness (s.d.)	Shannon diversity (s.d.)	Heterozygosity (s.d.)
Alice Springs (NT)	AS	1.42 (0.49)	1.23 (0.35)	1.19 (0.31)
Ardlethan (NSW)	AR	1.38 (0.48)	1.23 (0.34)	1.19 (0.30)
Batemans Bay (NSW)	BB	1.39 (0.49)	1.24 (0.34)	1.19 (0.31)
Bega Valley (NSW)	BV	1.42 (0.49)	1.24 (0.33)	1.19 (0.30)
Borroloola (NT)	BO	1.38 (0.49)	1.24 (0.34)	1.19 (0.31)
Bowen (QLD)	BW	1.40 (0.49)	1.24 (0.34)	1.19 (0.30)
Brisbane (QLD)	BR	1.52 (0.50)	1.25 (0.33)	1.19 (0.29)
Broome (WA)	BM	1.28 (0.45)	1.17 (0.32)	1.14 (0.29)
Cairns (QLD)	CR	1.26 (0.44)	1.20 (0.35)	1.17 (0.31)
Canberra (ACT)	CA	1.47 (0.50)	1.25 (0.33)	1.19 (0.29)
Cape Tribulation (QLD)	CT	1.49 (0.50)	1.25 (0.33)	1.19 (0.29)
Cape York (QLD)	CY	1.42 (0.49)	1.25 (0.34)	1.19 (0.30)
Cloncurry (QLD)	CL	1.34 (0.47)	1.22 (0.34)	1.17 (0.30)
Coen (QLD)	CO	1.41 (0.49)	1.24 (0.34)	1.19 (0.30)
Cooktown (QLD)	CK	1.41 (0.49)	1.24 (0.34)	1.19 (0.30)
Darwin (NT)	DA	1.37 (0.48)	1.23 (0.34)	1.19 (0.30)
Griffith (NSW)	GR	1.36 (0.48)	1.22 (0.34)	1.18 (0.30)
Hughenden (QLD)	HU	1.38 (0.49)	1.23 (0.34)	1.18 (0.30)
Katherine (NT)	KA	1.36 (0.48)	1.22 (0.34)	1.18 (0.30)
Kununurra (WA)	KU	1.37 (0.48)	1.22 (0.34)	1.18 (0.30)
Loyalty Island (PI)	LI	1.32 (0.47)	1.21 (0.34)	1.17 (0.30)
Mapoon (QLD)	MP	1.40 (0.49)	1.24 (0.34)	1.19 (0.30)
Mareeba (QLD)	MA	1.56 (0.50)	1.26 (0.32)	1.20 (0.29)
Mataranka (NT)	MK	1.38 (0.49)	1.23 (0.33)	1.18 (0.30)
Mt Isa (QLD)	MI	1.36 (0.48)	1.22 (0.34)	1.18 (0.30)
Narrabri (NSW)	NA	1.41 (0.49)	1.24 (0.34)	1.19 (0.30)
New Caledonia (PI)	NC	1.34 (0.48)	1.21 (0.33)	1.17 (0.30)
Rockhampton (QLD)	RO	1.45 (0.50)	1.25 (0.34)	1.19 (0.30)
Shepparton (VIC)	SH	1.39 (0.49)	1.23 (0.34)	1.18 (0.30)
Sydney (NSW)	SY	1.46 (0.50)	1.24 (0.33)	1.19 (0.29)
Tahiti (PI)	TH	1.26 (0.44)	1.18 (0.33)	1.15 (0.29)
Torrens Creek (QLD)	TC	1.40 (0.49)	1.24 (0.34)	1.19 (0.30)
Townsville (QLD)	TO	1.39 (0.49)	1.23 (0.33)	1.18 (0.30)
Utchee Creek (QLD)	UC	1.41 (0.49)	1.24 (0.34)	1.19 (0.30)
Weipa (QLD)	WP	1.33 (0.47)	1.22 (0.35)	1.18 (0.31)

ACT: Australian Capital Territory; **QLD:** Queensland; **NSW:** New South Wales; **PI:** Pacific Island; **WA:** Western Australia; s.d.: Standard deviation.

Table S3: Pairwise Fst values for populations in the N+E+D dataset. The lower triangle shows the Fst values between population pairs, and the upper triangle shows the p-value of each comparison. Significant comparisons are shown as red and non-significant ones are shown as **ns**.

	AS	RO	CT	MA	BR	BM	SY	DA	CA	BV	KU	NA	SH	UC	BB	GR	BO	KA	AR	MK	TC	CL	HU	MI	NC	LI	CO	CK	BW	TO	MP	CY	WP	CR
**AS**		0.00	0.00	0.00	0.00	0.00	0.00	0.00	0.00	0.00	0.00	0.00	0.00	0.00	0.00	0.00	0.00	0.00	0.00	0.00	0.00	0.00	0.00	0.00	0.00	0.00	0.00	0.00	0.00	0.00	0.00	0.00	0.00	0.00
**RO**	0.11		0.00	ns	ns	0.00	0.00	0.00	0.00	0.00	0.00	ns	0.00	0.00	0.00	0.00	0.00	0.00	0.00	0.00	ns	0.00	ns	0.00	0.00	0.00	ns	ns	ns	ns	0.00	0.00	ns	ns
**CT**	0.12	0.01		0.00	0.00	0.00	0.00	0.00	0.00	0.00	0.00	0.00	0.00	0.01	0.00	0.00	0.00	0.00	0.00	0.00	0.01	0.00	0.00	0.00	0.00	0.00	0.01	ns	0.00	0.00	0.00	0.00	0.04	ns
**MA**	0.11	0.00	0.01		0.02	0.00	0.00	0.00	0.00	0.00	0.00	ns	0.00	0.02	0.00	0.00	0.00	0.00	0.00	0.00	ns	0.00	0.00	0.00	0.00	0.00	0.04	0.00	0.01	0.00	0.00	0.00	ns	ns
**BR**	0.11	0.00	0.01	0.00		0.00	0.00	0.00	0.00	0.00	0.00	ns	0.00	ns	0.00	0.00	0.00	0.00	0.00	0.00	ns	0.00	0.00	0.00	0.00	0.00	0.00	0.03	0.04	0.00	0.00	0.00	ns	ns
**BM**	0.27	0.22	0.22	0.21	0.22		0.00	0.00	0.00	0.00	0.00	0.00	0.00	0.00	0.00	0.00	0.00	0.00	0.00	0.00	0.00	0.00	0.00	0.00	0.00	0.00	0.00	0.00	0.00	0.00	0.00	0.00	0.00	0.00
**SY**	0.12	0.01	0.01	0.01	0.01	0.24		0.00	0.00	0.00	0.00	0.00	0.00	0.00	0.00	0.00	0.00	0.00	0.00	0.00	0.02	0.00	0.00	0.00	0.00	0.00	0.00	0.00	0.04	0.00	0.00	0.00	0.04	0.00
**DA**	0.13	0.05	0.05	0.05	0.06	0.19	0.06		0.00	0.00	0.00	0.00	0.00	0.00	0.00	0.00	0.00	0.00	0.00	0.00	0.00	0.00	0.00	0.00	0.00	0.00	0.00	0.00	0.00	0.00	0.00	0.00	0.00	0.00
**CA**	0.12	0.01	0.02	0.01	0.01	0.24	0.01	0.06		0.00	0.00	0.00	0.00	0.00	0.00	0.00	0.00	0.00	0.00	0.00	0.00	0.00	0.00	0.00	0.00	0.00	0.00	0.00	0.00	0.00	0.00	0.00	0.01	0.00
**BV**	0.13	0.02	0.03	0.02	0.02	0.25	0.03	0.09	0.03		0.00	0.00	0.00	0.00	0.00	0.00	0.00	0.00	0.00	0.00	0.00	0.00	0.00	0.00	0.00	0.00	0.00	0.00	0.00	0.00	0.00	0.00	0.00	0.00
**KU**	0.19	0.10	0.10	0.10	0.11	0.20	0.12	0.05	0.12	0.13		0.00	0.00	0.00	0.00	0.00	0.00	0.00	0.00	0.01	0.00	0.00	0.00	0.00	0.00	0.00	0.00	0.00	0.00	0.00	0.00	0.00	0.00	0.00
**NA**	0.12	0.00	0.01	0.00	0.00	0.24	0.01	0.06	0.01	0.03	0.11		0.00	0.01	0.00	0.00	0.00	0.00	0.00	0.00	ns	0.00	0.03	0.00	0.00	0.00	0.00	0.03	ns	0.00	0.00	0.00	ns	0.04
**SH**	0.13	0.02	0.03	0.02	0.02	0.26	0.03	0.08	0.02	0.04	0.13	0.02		0.00	0.00	0.00	0.00	0.00	0.00	0.00	0.00	0.00	0.00	0.00	0.00	0.00	0.00	0.00	0.00	0.00	0.00	0.00	0.00	0.00
**UC**	0.12	0.01	0.01	0.00	0.00	0.24	0.01	0.05	0.02	0.03	0.11	0.01	0.03		0.00	0.00	0.00	0.00	0.00	0.00	0.01	0.00	0.00	0.00	0.00	0.00	0.00	0.01	0.02	0.00	0.00	0.00	ns	ns
**BB**	0.12	0.01	0.02	0.01	0.01	0.25	0.01	0.06	0.01	0.02	0.12	0.01	0.03	0.02		0.00	0.00	0.00	0.00	0.00	0.01	0.00	0.00	0.00	0.00	0.00	0.00	0.00	0.00	0.00	0.00	0.00	0.00	0.03
**GR**	0.15	0.04	0.04	0.03	0.04	0.27	0.04	0.09	0.03	0.04	0.14	0.03	0.03	0.04	0.03		0.00	0.00	0.00	0.00	0.00	0.00	0.00	0.00	0.00	0.00	0.00	0.00	0.00	0.00	0.00	0.00	0.00	0.00
**BO**	0.15	0.06	0.06	0.06	0.07	0.18	0.07	0.03	0.07	0.09	0.02	0.06	0.08	0.07	0.07	0.09		0.00	0.00	0.00	0.00	0.00	0.00	0.00	0.00	0.00	0.00	0.00	0.00	0.00	0.00	0.00	0.00	0.00
**KA**	0.18	0.09	0.09	0.09	0.10	0.20	0.12	0.04	0.11	0.13	0.01	0.10	0.12	0.10	0.12	0.14	0.01		0.00	ns	0.00	0.00	0.00	0.00	0.00	0.00	0.00	0.00	0.00	0.00	0.00	0.00	0.00	0.00
**AR**	0.13	0.02	0.02	0.02	0.02	0.27	0.02	0.08	0.02	0.03	0.13	0.02	0.03	0.02	0.02	0.03	0.08	0.13		0.00	0.00	0.00	0.00	0.00	0.00	0.00	0.00	0.00	0.00	0.00	0.00	0.00	0.00	0.00
**MK**	0.18	0.09	0.09	0.09	0.10	0.19	0.11	0.04	0.11	0.12	0.01	0.10	0.13	0.10	0.12	0.14	0.01	0.00	0.13		0.00	0.00	0.00	0.00	0.00	0.00	0.00	0.00	0.00	0.00	0.00	0.00	0.00	0.00
**TC**	0.12	0.00	0.01	0.00	0.00	0.24	0.01	0.05	0.01	0.02	0.10	0.00	0.02	0.01	0.01	0.04	0.06	0.10	0.01	0.09		0.00	0.04	0.00	0.00	0.00	ns	ns	ns	ns	0.00	ns	ns	ns
**CL**	0.16	0.05	0.06	0.05	0.06	0.27	0.06	0.09	0.07	0.08	0.13	0.06	0.07	0.07	0.06	0.09	0.08	0.13	0.08	0.13	0.05		0.00	0.00	0.00	0.00	0.00	0.00	0.00	0.00	0.00	0.00	0.00	0.00
**HU**	0.12	0.00	0.01	0.01	0.01	0.23	0.01	0.06	0.01	0.03	0.10	0.01	0.02	0.01	0.01	0.04	0.05	0.09	0.02	0.09	0.01	0.06		0.00	0.00	0.00	ns	ns	0.01	ns	0.00	ns	ns	ns
**MI**	0.12	0.03	0.04	0.03	0.03	0.26	0.04	0.06	0.04	0.06	0.12	0.04	0.05	0.04	0.04	0.07	0.07	0.12	0.06	0.11	0.03	0.07	0.04		0.00	0.00	0.00	0.00	0.00	0.00	0.00	0.00	0.00	0.00
**NC**	0.19	0.08	0.09	0.08	0.08	0.32	0.09	0.15	0.09	0.10	0.18	0.08	0.10	0.09	0.09	0.12	0.15	0.19	0.11	0.17	0.09	0.14	0.08	0.12		0.00	0.00	0.00	0.00	0.00	0.00	0.00	0.00	0.00
**LI**	0.20	0.10	0.10	0.09	0.09	0.32	0.10	0.15	0.11	0.11	0.20	0.10	0.11	0.10	0.10	0.13	0.17	0.20	0.11	0.19	0.10	0.16	0.11	0.13	0.02		0.00	0.00	0.00	0.00	0.00	0.00	0.00	0.00
**CO**	0.12	0.01	0.01	0.00	0.01	0.23	0.02	0.05	0.02	0.03	0.08	0.01	0.02	0.01	0.01	0.04	0.03	0.07	0.02	0.07	0.00	0.05	0.00	0.03	0.09	0.11		ns	0.01	ns	ns	ns	ns	ns
**CK**	0.11	0.00	0.00	0.01	0.01	0.23	0.01	0.04	0.02	0.02	0.08	0.01	0.03	0.01	0.01	0.04	0.04	0.07	0.03	0.07	0.00	0.06	0.01	0.03	0.09	0.10	0.00		0.03	ns	ns	ns	ns	ns
**BW**	0.11	0.00	0.01	0.01	0.01	0.24	0.01	0.06	0.01	0.03	0.11	0.00	0.02	0.01	0.02	0.04	0.06	0.10	0.02	0.10	0.00	0.06	0.01	0.04	0.09	0.11	0.01	0.01		0.00	0.00	0.00	ns	0.03
**TO**	0.13	0.00	0.02	0.01	0.01	0.25	0.01	0.06	0.02	0.02	0.11	0.01	0.03	0.02	0.02	0.04	0.05	0.10	0.03	0.09	0.01	0.06	0.00	0.04	0.08	0.10	0.00	0.01	0.01		0.00	0.03	ns	ns
**MP**	0.13	0.01	0.01	0.01	0.02	0.21	0.02	0.03	0.03	0.04	0.06	0.02	0.04	0.02	0.03	0.05	0.02	0.05	0.04	0.05	0.01	0.06	0.02	0.04	0.10	0.11	0.00	0.00	0.01	0.02		0.03	ns	ns
**CY**	0.12	0.01	0.01	0.01	0.01	0.22	0.02	0.04	0.02	0.03	0.07	0.01	0.03	0.01	0.02	0.04	0.03	0.06	0.03	0.06	0.01	0.05	0.00	0.04	0.09	0.10	0.00	0.00	0.01	0.01	0.01		ns	ns
**WP**	0.12	0.00	0.01	0.00	0.00	0.24	0.01	0.04	0.01	0.02	0.09	0.01	0.02	0.01	0.01	0.04	0.04	0.07	0.03	0.08	0.00	0.05	0.00	0.03	0.09	0.11	-0.01	-0.01	0.00	0.00	0.00	0.00		ns
**CR**	0.13	0.01	0.01	0.01	0.01	0.27	0.02	0.05	0.02	0.04	0.09	0.01	0.02	0.01	0.01	0.05	0.04	0.08	0.03	0.08	0.00	0.06	0.01	0.04	0.10	0.12	0.00	0.00	0.01	0.01	0.01	0.00	-0.01	
**TH**	0.26	0.17	0.18	0.16	0.17	0.39	0.18	0.23	0.18	0.19	0.26	0.18	0.20	0.18	0.19	0.21	0.23	0.27	0.20	0.25	0.19	0.23	0.18	0.21	0.14	0.15	0.19	0.19	0.19	0.18	0.19	0.17	0.19	0.22

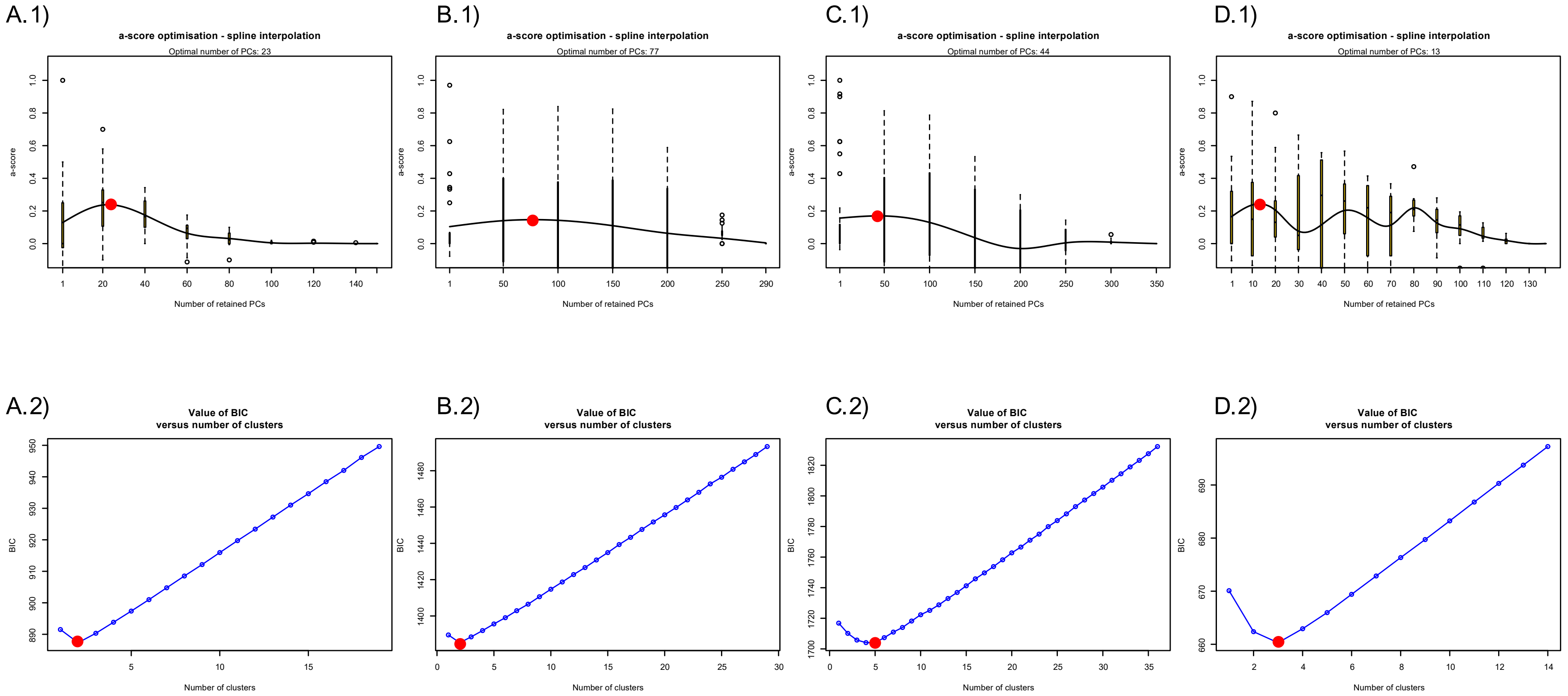

Figure S1. Alpha score analysis and Bayesian Information Criteria (BIC) used to obtain the optimal number of PCs and discriminant functions to retain in the DAPC analysis of the (A) N (B) N+E (C) N+E+D and (D) temporal datasets. Red circles indicate the optimal number of PCs and discriminant functions retained for each dataset.

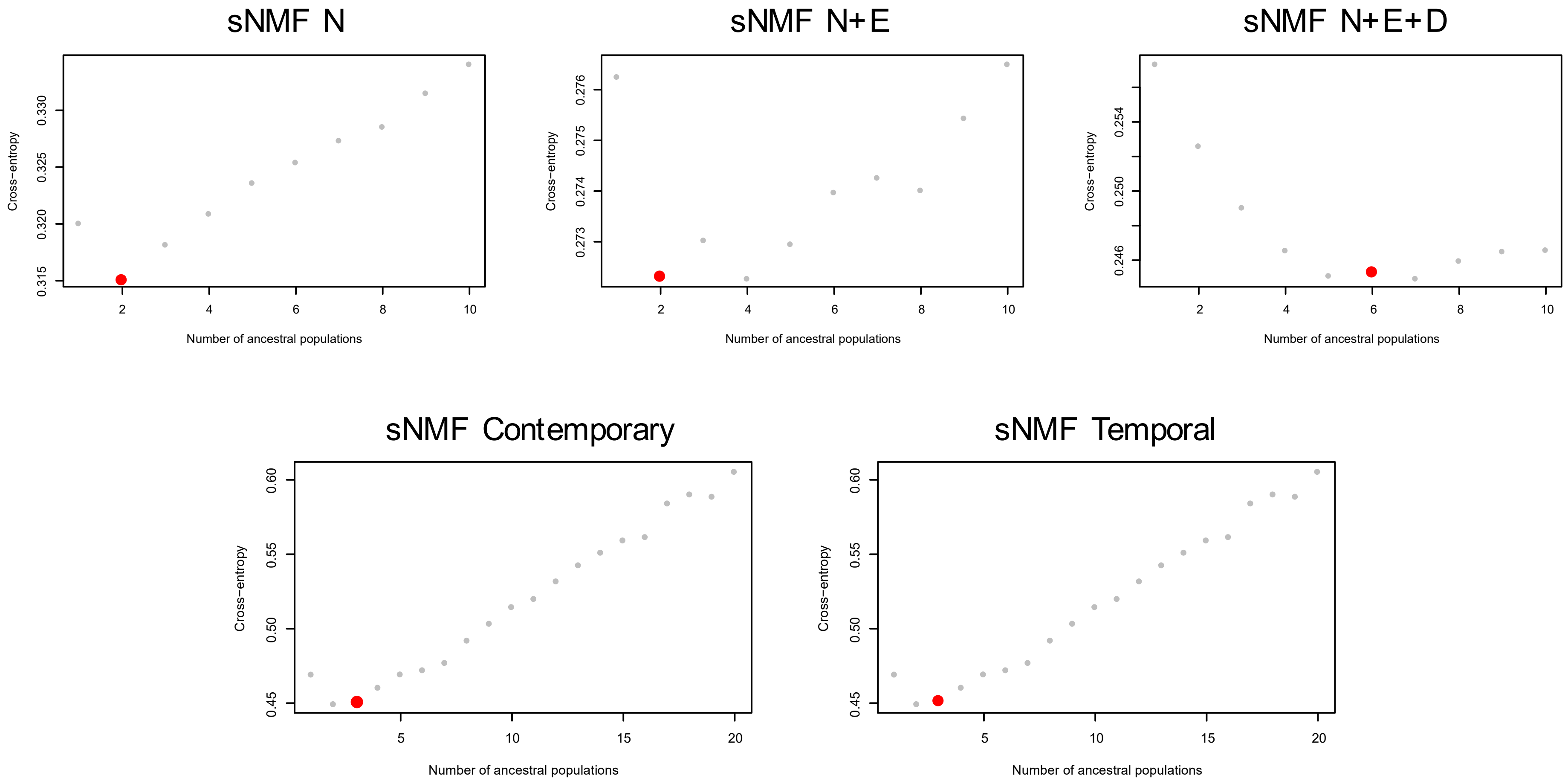

Figure S2. Minimal cross-entropy for each number of ancestral populations (K) from 1 to 10. Red circles indicate the value of K that best represents the population history for each dataset.

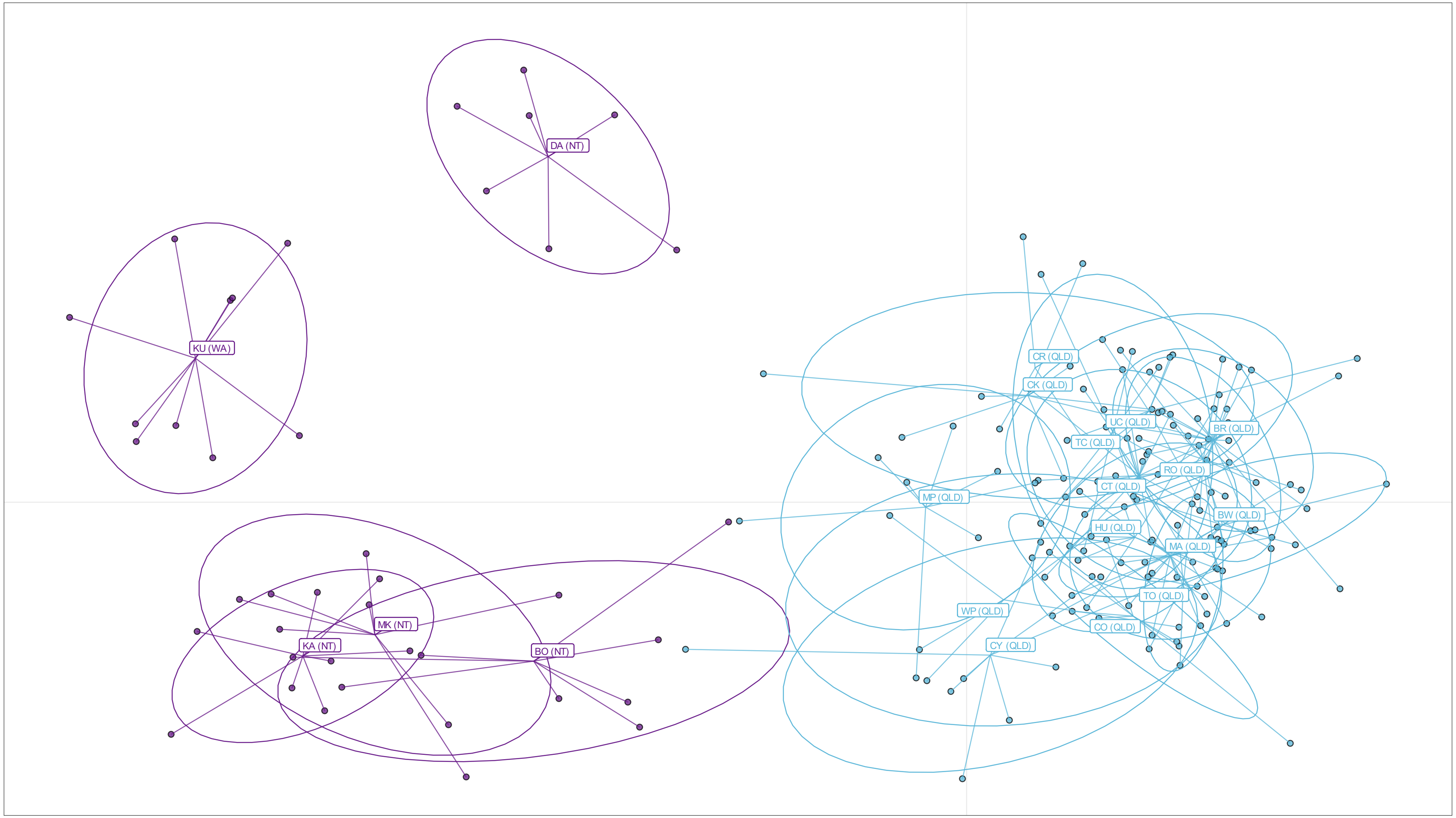

Figure S3. Discriminant Analysis of Principal Components of individuals from the Qfly and NTfly native range. As per Fig. 2, colours are representative of the admixture proportions of every individual estimated with sNMF. Blue: QLD ancestry; and Purple: NTfly ancestry.

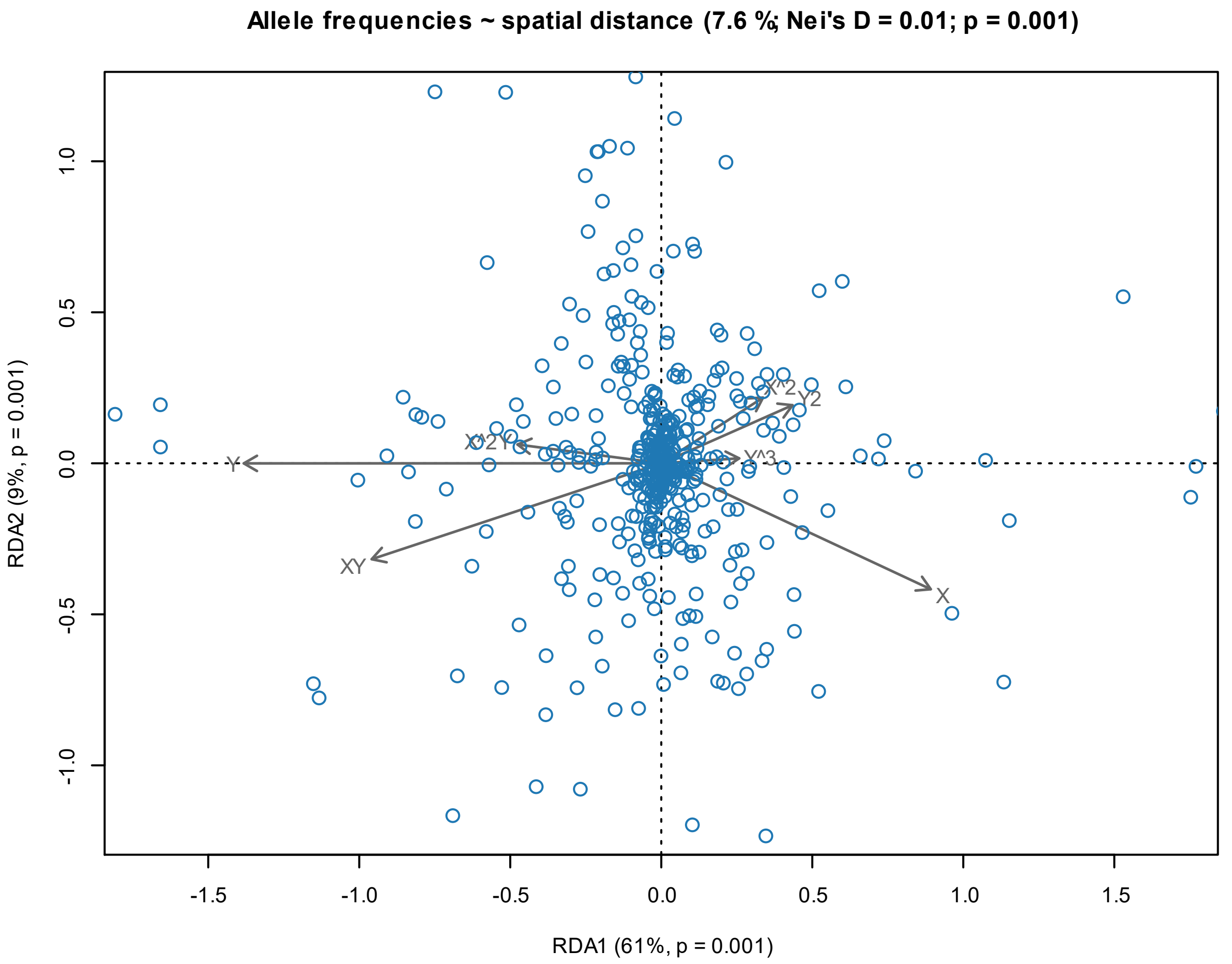

Figure S4: Redundancy analysis (RDA) of allele frequencies against polynomial functions of latitude and longitude (maximum power 2) for each population in the native and expanded range. Alleles are indicated by circles, and the arrows' length and direction correspond to the variance that can be explained by the distance polynomials. The directions of the arrow indicate increasing effects of longitude, latitude or their polynomials on allele frequency differences.

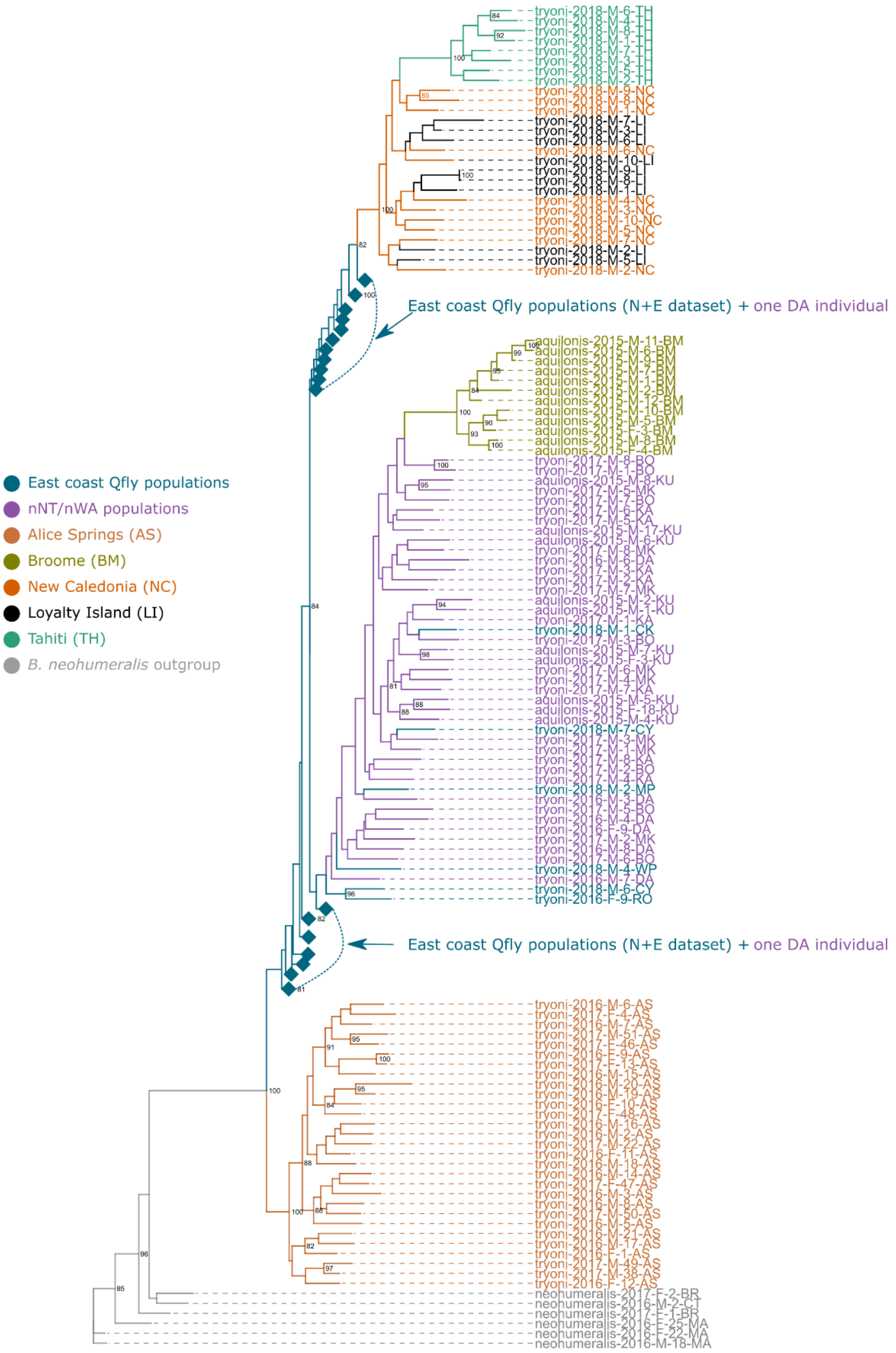

Figure S5. Maximum likelihood phylogenetic tree for the N+E+D dataset. IQ-tree [58] was run with the TVM+F+I+G4 DNA substitution model as it resulted in the lowest BIC out of 286 DNA substitution models tested via the model finding algorithm (-m MFP) [59]. Branch support was assessed via bootstrapping with 1000 repetitions. To reduce the impact of model violation in the estimated bootstrapped values we optimised each bootstrap tree by using a hill-climbing nearest neighbour interchange search-based algorithm [60]. The final consensus tree was visualised with the *ggtree* [61] packages in R, only showing bootstraps values over 80%. Population abbreviations are as per Fig. 1.
